# Susceptible and infectious states for both vector and host in a dynamic pathogen–vector–host system

**DOI:** 10.1098/rspb.2023.2293

**Published:** 2024-01-10

**Authors:** Zachary S. Lamas, Maiya Krichton, Eugene V. Ryabov, David J. Hawthorne, Jay D. Evans

**Affiliations:** ^1^ Bee Research Laboratory, United States Department of Agriculture—Agricultural Research Service, Beltsville 06415, MD, USA; ^2^ Department of Entomology, University of Maryland, College Park 20742-5031, MD, USA; ^3^ The James Hutton Institute, Invergowrie, Dundee, UK

**Keywords:** disease transmission, honeybees, *Varroa*

## Abstract

Deformed wing virus (DWV) is a resurgent insect pathogen of honeybees that is efficiently transmitted by vectors and through host social contact. Continual transmission of DWV between hosts and vectors is required to maintain the pathogen within the population, and this vector–host–pathogen system offers unique disease transmission dynamics for pathogen maintenance between vectors and a social host. In a series of experiments, we measured vector–vector, host–host and host–vector transmission routes and show how these maintain DWV in honeybee populations. We found co-infestations on shared hosts allowed for movement of DWV from mite to mite. Additionally, two social behaviours of the honeybee, trophallaxis and cannibalization of pupae, provide routes for horizontal transmission from bee to bee. Circulation of the virus solely among hosts through communicable modes provides a reservoir of DWV for naïve *Varroa* to acquire and subsequently vector the pathogen. Our findings illustrate the importance of community transmission between hosts and vector transmission. We use these results to highlight the key avenues used by DWV during maintenance and infection and point to similarities with a handful of other infectious diseases of zoonotic and medical importance.

## 1. Introduction

Continual transmission between infectious and susceptible individuals is essential for the persistence of a pathogen in a population [1]. Infectious individuals, aptly called maintenance hosts, maintain an infectious disease within a population, serving as a reservoir of disease for both conspecifics and vectors who feed upon them.

Numerous theoretical models have been developed to describe the transmission dynamics of infectious diseases [2,3], encompassing both vector-borne and contagious diseases. These models rely on complete and accurate estimates of routes and parameters of transmission, which may be lacking in emerging diseases. One such disease is caused by the widespread pathogen deformed wing virus (DWV). Once rarely observed in the host *Apis mellifera* (hereafter referred to as honeybee), DWV is now highly prevalent in managed and wild colonies of honeybees [4], and has been detected in more than 65 arthropod species, presumably an indication of spillover from infected honeybees [5]. DWV is an RNA virus from the *Iflaviridae* family transmitted through vertical and horizontal routes between honeybees. As an infectious disease, DWV became a vector-borne pathogen in global honeybee populations [4] once *Varroa destructor*, an ectoparasitic mite, became established on *Apis mellifera* after jumping hosts from *Apis cerana* [6]*.* Impacts of DWV garnered global attention when severe losses in managed and feral honeybee colonies were reported in 2006 in North America [7], leading to intensive research on DWV and its impacts [5].

DWV is transmitted via multiple communicable modes among the thousands of members found in honeybee colonies [8]. Mockel *et al.* showed through artificial feeding that adult workers could develop covert (subclinical) infection after oral exposure to the virus [9]. Consumption of infectious pupae by adult bees followed by transmission through trophallaxis between nest-mates was shown to be an efficient route for transmission, which also resulted in covert infection [10]. Brood rearing is an essential function in a honeybee colony. Yue & Genersch detected DWV in larval food, suggesting brood rearing may be a route of infection for developing bees [11]. In addition, infection in developing brood has been shown to originate through the queen via vertical transmission. Amiri *et al.* demonstrated experimentally that one-third of colonies had eggs infected with DWV. The virus was present on the surface of the eggs indicating transovum transmission [12]. Direct infection of queens has been demonstrated experimentally through artificial insemination of virgin queens and in field studies through natural mating [13,14]. Importantly, covert infections arising from the queen and passing vertically to her thousands of offspring could lead to sustained transmission within the colony, a key parameter in the maintenance of DWV. De Miranda and Fries postulated that infections through this route would influence *Varroa*-mediated and fecal-oral-cannibalization transmission routes by potentially infecting a large swath of bees [15].

*Varroa* feed on bees both during their reproductive phase on developing larvae, and during a non-reproductive phase on adult bees [16–18]. Feeding bouts during both periods offer opportunities for vector-mediated transmission to hosts. Overt (clinical) infections are most evident in the form of crippled wings, shortened abdomens, and adults with motor-paralysis resulting from transmission during larval development [7,19]. Subclinical infections occur when bees are parasitized during development but do not develop physical deformities, and are often associated with lower viral loads [7,20]. Ball *et al*. showed mite invasions on developing pupae led to DWV infections in the hosts, while Mockel *et al*. showed infections in adult bees could occur through artificial injections [9]. The magnitude at which *Varroa destructor* can change the pathogen profile of its host was most notably seen when *Varroa* moved into the Hawaiian archipelago. DWV loads were observed to amplify in host populations once *Varroa* moved into this new, non-endemic region [6].

*Varroa* can be infectious with a virus, or naive, without a virus [21,22]. Bees harboring high levels of DWV may serve as important maintenance hosts, allowing naïve *Varroa* which feed upon them to acquire and subsequently transmit the pathogen. Bee-to-mite transmission occurs when *Varroa* feed on an already infected host which has developed a high level of infection prior to the *Varroa*'s feeding [23]. Bowen-Walker *et al*. inferred this from high levels of DWV in *Varroa* collected from infectious bees with crippled wings [24]. However, in such a dynamic vector–host–pathogen system, multiple potential transmission routes could facilitate host-to-vector acquisition of the pathogen.

For example, indirect mite-mite transmission has not yet been observed but could potentially occur during both *Varroa*'s reproductive phase or during the non-reproductive phase when two or more *Varroa* share the same larval or adult bee host, simultaneously or concurrently. Multiple infestations, while not as common as single infestations, may still be an important parameter in the maintenance and spread of DWV between mites [25]. Similar features are important in the maintenance of pathogens in other parasites and vectors, most notably with cofeeding ticks [26–28]. Both mite-mite and bee-mite transmission routes could be important in the maintenance of DWV in the *Varroa* population.

In a series of experiments, we asked if transmission occurs indirectly from mite-to-mite on shared hosts, and through bee-to-mite routes. When examining bee-to-mite transmission routes, we asked if circulation of the virus through social interactions amongst hosts can influence acquisition and subsequent vector-borne transmission of the virus by the vector. Specifically, we assessed whether adult bees which acquired DWV through trophallaxis could be infectious to naïve *Varroa.* In a similar manner, we studied how cannibalization of infectious pupae by adult bees supported acquisition of the virus by *Varroa* that fed upon those adult bees. When examining indirect transmission between mites, we examined transmission of DWV to naïve mites through a shared host with an infectious mite.

Although many of these routes and interactions have been assumed for years, there is scant quantitative evidence. We describe here the integration between communicable and vector-borne transmission modes; social behaviours of the host species aid in transmission, acquisition and future vectoring of the pathogen by the *Varroa*. In the most notable example, hygienic removal of diseased pupae by adult bees, a form of social defense in honeybees, aids in the spread and persistence of DWV in hosts and *Varroa*. These integrated modes help explain the devastating spread and impacts of RNA viruses on honeybee health.

## 2. Material and methods

### (a) Construction of custom cages

Custom laboratory arenas were constructed from acrylic sheets so that bees could be physically isolated in adjoining, but divided chambers. The divider was a solid piece of acrylic with a 0.875-inch hole cut in the centre. The hole was sealed with 0.125-inch hardware cloth. This served as a trophallaxis port in which bees could orally exchange food with limited physical contact. A 0.125-inch fibreglass mesh screen was used for ventilation at the end of each chamber. The interior dimensions of each cage were 2.5″ × 2.5″ × 2.75″.

### (b) Viral inoculum

A clone-derived variant of DWV-A tagged with nanoluciferase (NLuc) gene was used throughout the trials [29]. This virus has two inserted genetic tags in its genomic RNA which allow for reliable detection and make it possible to distinguish the clone-derived virus from wild-type variants. First, specific flanking sequences allow the quantification by RT-qPCR of both clone-derived and native virus. Second, a rare-cut site for the PacI restriction endonuclease distinguishes the clone-derived product by restriction enzyme screens [29]. In this way, dual verification of the presence of the tagged virus was achieved. For the rest of the document inoculum refers to the DWV-A NLuc virus. Stock samples were provided by Evans and Ryabov and maintained at the USDA-ARS Bee Research Laboratory in Beltsville, Maryland.

### (c) Collection and maintenance of *Varroa* and delivery of viral inoculum

*Varroa destructor* mites (hereafter *Varroa*) were sourced and hand collected from a heavily infested colony not showing overt signs clinical disease. Adult bees were inspected and those with *Varroa* in their feeding positions were maintained in a ventilated cage [23]. Bees and *Varroa* were returned to the laboratory and maintained in an incubator at 34°C and 40% relative humidity. Once returned to the laboratory *Varroa* were transferred to pupae in a size 0 gel cap (20 mm length × 8 mm diameter), and incubated for 12 h at 34°C and 40% humidity). These *Varroa* were serially passaged by exchanging new pupal hosts as a means of reducing potential viral loads carried by these *Varroa* [30]. At the last passage *Varroa* were randomly assigned to a control (PBS injected) or experimental (viral injected). Pupae were injected with 1 ul of either PBS or viral inoculum (10^7^ GE). These pupae were injected 12 h prior to the introduction of *Varroa*. *Varroa* were transferred to pupae were transferred into a size 0 gel cap, and returned to the incubator to feed for 24 h (total 36 h post injection). After feeding for 24 h on an injected pupa, *Varroa* were transferred to experimental arenas.

### (d) Marking of *Varroa*

*Varroa* were marked in these trials with a small amount of paint applied dorsal-distally directly onto the carapace. Paint was applied with a 0000 fine tip paintbrush (Javis: 4/0 nylon, England) with a small downward strike moving posteriorly from the dorsal tip of the *Varroa*. Paint from fine-tipped oil-based permanent markers were used. Paint was ejected from the tip of the marker onto a plastic surface until a pool of paint formed. The tip of the brush was then dabbed against the surface of this pool. In this way, a small amount of paint could be applied to the surface of a *Varroa*. Colours were used to identify *Varroa* by group. For example, *Varroa* painted blue were part of the viral group, clearly identifying them differently than the *Varroa* from the control group which were painted orange. The researcher could observe, track and recollect multiple *Varroa* co-housed on the same group of bees.

### (e) Molecular preparation and qPCR

RNA was extracted from individual bees and mites using the TRIzol method (Invitrogen), starting with 1000 ul and 200 ul, respectively of Tri reagent and chloroform. Extracted RNA was used to produce cDNA using BioRad iScript reverse transcriptase according to manufacturer specifications. Total viral cDNA was quantified using real-time qPCR and a 10-fold dilution series of prepared standards exactly as described in Posada-Florez *et al*. [10]. Detection of the PacI site in the 5′ region of DWV-ANluc RNA was performed through digestion of the RT-PCR fragment corresponding to the positions 30–1266 nt of the virus genome with PacI and visualization of the products by agarose gel electrophoresis [10].

### (f) Experiment 1: can communicable transmission of DWV between honeybees amplify future acquisition and vectoring of the pathogen?

Transmission studies were carried out in custom trophallaxis arenas (described above). Three experimental groups were created for this experiment: no *Varroa*, *Varroa* and *Varroa + virus* (*n* = 5 arenas per group; table 1).

**Table 1.** Description of experimental groups in Experiment 1.

group name	description
no *Varroa*	donor bees were never parasitized by mites
*Varroa*	donor bees were parasitized by mites
*Varroa +* virus	donor bees were parasitized by mites which were given an additional virus

**Figure 1.**
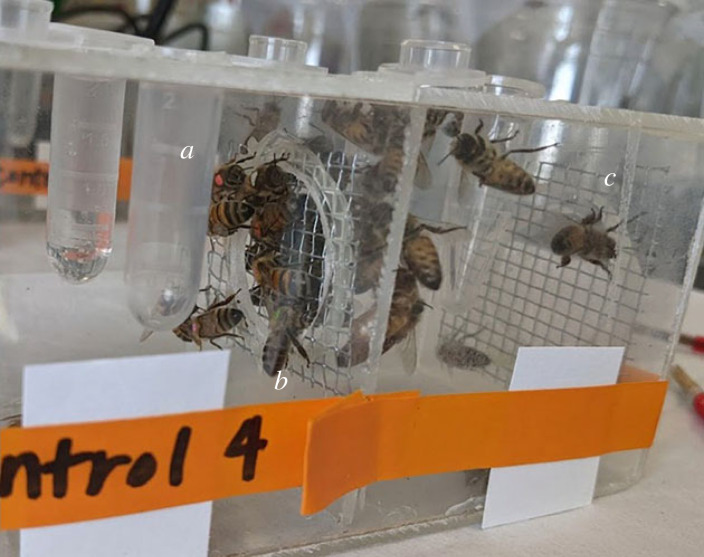
(*a*) Donor bee compartment. (*b*) Mesh screen divider. (*c*) Recipient bee compartment.

In the *no Varroa* group donor bees were not exposed to mites. In the *Varroa* group, donor bees were exposed to *Varroa* without additional viruses given, and lastly the *Varroa* + virus group consisted of donor bees exposed to *Varroa* with additional virus given to the *Varroa* (see above for delivery of viral inoculum). Donor bees were transferred to trophallaxis arenas after *Varroa* were removed from their body (figure 1*a*). Bees were added to the arenas over the course of 4 days and incubated for a total of 7 days. Bees were checked twice daily for the presence of *Varroa*, and to record bee mortality.

On day 8 of the trial, newly emerged workers were added to the ‘recipient’ compartment of the custom trophallaxis arenas (figure 1*c*). Recipient bees were thoroughly inspected for *Varroa* at emergence before introduction into the custom arenas. Trophallaxis was encouraged by removing sucrose feeders from the recipient's side by removing sucrose feeders for 4 h, after which bees were checked for *Varroa*, and then feeders were returned. This continued for 8 more days (15 total days since first addition of donor bees).

**Figure 2.**
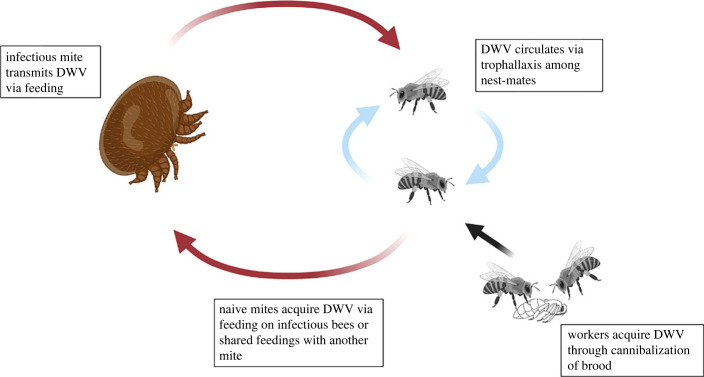
Model of DWV circulation among worker bees and *Varroa.* Red arrows represent vectoring of DWV to bees or acquisition of DWV to *Varroa* via infectious bees. Blue arrows represent circulation of DWV through trophallaxis. Black arrow represents infection from cannibalization. Complex transmission dynamics theoretically supports social behaviours maintaining pathogen for subsequent vector acquisition.

On day 16, donor bees were removed from all 15 experimental arenas and stored at −80°C, leaving only recipient bees in the trophallaxis arenas. Recipient bees were thoroughly inspected for *Varroa* one final time (no *Varroa* were found in any of the arenas). A total of 101 *Varroa* were used for the remainder of this experiment. Four *Varroa* were removed at random and stored at −80°C for RNA extraction. These mites served to verify the Nluc RNA sequence was not present in mites prior to the trial. The remaining 97 *Varroa* were placed onto recipient bees across the three experimental groups. Fresh *Varroa* were then added to each arena of recipient bees (*n* = 97). *Varroa* were allowed to feed on adult recipient bees for 4 days. At the end of the 4-day period, a subcollection of *Varroa* from each group were removed and immediately saved at −80°C (total *n* = 33). The remaining *Varroa* still in the study (*n* = 39) were transferred from their adult bee host to a white eyed pupa in size 0 gel caps, incubated for 6 days and then preserved at −80°C for later RNA extraction (figure 2).

### (g) Experiment 2: do *Varroa* acquire virus when feeding upon adult bees which previously cannibalized pupae?

Experimental pupae used for cannibalization by adult worker bees were procured using established methods [10], and then introduced to cages of 30 adult bees. Cages were randomly assigned to one of three treatment groups: no pupae, pupae, pupae + virus (from here on referred to as: negative control, control, experimental). One pupa was removed from the −80°C freezer for each experimental cage, thawed and immediately fed to the bees. This was repeated on the following day, after which bees were incubated for an additional 9 days (11 days total since first introduction). *Varroa* were then introduced into each experimental cage and allowed to feed upon these adult bees for 4 days, after which *Varroa* were individually removed and introduced into gel caps with a white-eyed pupa (non-injected). *Varroa* were allowed to feed on pupae for 48 h, at which point the *Varroa* were removed and stored at −80°C. Pupae were incubated for 4 more days and then stored at −80°C.

### (h) Experiment 3: does DWV transmit indirectly from mite to mite on shared honeybee hosts?

In two separate experiments, indirect transmission of tagged virus from mite to mite through a shared host was tested. In the first experiment transmission from mite-mite was tested on shared pupal hosts, while in the second experiment transmission from mite-mite was tested on shared adult worker bees.

Indirect mite-mite transmission on a shared pupal host was tested by inserting *Varroa* into recently capped larval cells similar to Frey *et al.* [31]. In short, a frame of recently capped worker brood was removed from a colony without any *Varroa* detections and brought to the laboratory. The corner of a cell capping was lifted with a razor blade, and a *Varroa* was gently introduced inside the cell. *Varroa* were introduced with a Chinese grafting tool, inserting the *Varroa* into the opening of the torn cell. *Varroa* were coaxed into the cell by prodding them with an additional grafting tool held in the other hand. A second *Varroa* was immediately introduced in the same manner into the brood cell, and then the cell capping was promptly sealed. Cell cappings were resealed by pressing the torn capping back down, and using another cell capping as a ‘bandage’. This was done by removing an entire cell capping with a pair of tweezers, flipping it over, and firmly pressing the waxed side (silk side up) against the ‘wound’ of the experimental cell. If a cell was damaged too greatly to be re-sealed in this way, then it was sealed with a size 6 gel cap.

Two *Varroa* were introduced into each cell as pairs. These pairs were either control × control, or control × virus-exposed (viral). *Varroa* were paint marked as described above. Experimental cells were numbered in chronological order of insertion. Geographical reference points were placed on the surface of the frame to locate experimental cells.

*Varroa* and pupal hosts were recovered 9 days after insertion. On day 9, brood cells containing the experimental *Varroa* were excavated. The cell cappings were removed with a pair of tweezers, pupae were removed and saved at −80°C. *Varroa* were collected from the cells using a Chinese grafting tool, and then placed into a size 0 gel cap with a pink-eyed pupa. Pupae and *Varroa* were incubated for 48 h, at which time *Varroa* were removed and stored at −80°C until RNA extraction. Pupae were returned to the incubator for another 4 days, after which samples were preserved at −80°C.

Indirect mite-mite transmission on a shared adult bee host was tested. Pairs of *Varroa* were introduced into arenas with adult bees. Arenas were established in the same manner as previously published methods [23]*.* The feeding locations of each mite were checked 2 h after introduction into the arenas and then every 12 h thereafter for 10 continuous days. *Varroa* and adult bees were marked with oil-based sharpie markers as described above so that each individual could be uniquely identified. Daily *Varroa* and host mortality was recorded. All samples were saved at −80°C until extraction.

### (i) Statistical analysis

All statistical analysis were performed in RStudio using BaseR and associated packages. For all trials, count data were used to report the observed number of infectious bees or *Varroa* over the number of non-infectious bees or *Varroa* in a trial. In order to test viral load differences in exposed and non-exposed *Varroa* and bees to the NLuc virus an ANOVA was performed to see if there were significant differences between the groups. The residuals of data were visualized and then tested for normality using the Shapiro–Wilk test. DWV-A titres were compared across groups using an ANOVA. When the assumptions of normality were not met with NLuc titres a non-parametric Kruskal–Wallis ANOVA was used. Statistical significance was set at *p* < 0.05.

## 3. Results

### (a) Experiment 1: can communicable transmission of DWV between honeybees amplify future acquisition and vectoring of the pathogen?

**Figure 3.**
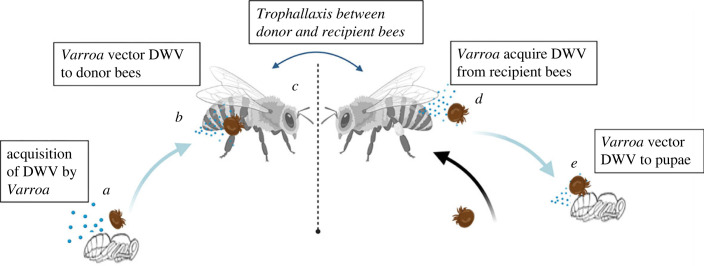
Virus transmission steps in Experiment 1. (*a*) *Varroa* acquired DWV-A with NLuc- and PacI-tagged genome from a pupa artificially infected with the tagged virus. (*b*) The viruliferous *Varroa* then successfully vectored the pathogen to donor bee hosts, who subsequently (*c*) transmitted it through trophallaxis to naïve recipient bees unexposed to original *Varroa*. (*d*) New *Varroa* were introduced into the system which acquired the pathogen (12/41, 29.3%) from the recipient bees infected through trophallaxis, and then (*e*) vectored it to new recipient pupal hosts (3/17, 17.6%). Detections of the tagged virus in *Varroa* mites was accomplished through the *Pac*I digestion of the RT-PCR product containing the tagged region of DWV genome and visualization of the products by agarose gel electrophoresis.

*Varroa* acquired DWV by feeding on recipient bees which had become infected through an oral transmission route via donor nest-mates (figure 3). Of the 97 mites used in this experiment, 25 died prior to the end of the trial.

Of the 41 *Varroa* recollected from the *Varroa +* virus group, 12 (29.3%) were positive for the tagged virus, while the tagged virus was not detected in *Varroa* that were exposed to either the negative control or control groups (0/29). Of the *Varroa* which reported detections, qPCR results showed low levels of the tagged virus (mean ± s.d. = 4.69 log_10_ ± 0.33, *n* = 12). Levels of natural (non-tagged) DWV-A were not significantly different between *Varroa* that were exposed to experimental or control bees (AOV, *F*_1,68_ = 1.885, *p* = 0.174). In fact, nearly all mites sampled in this trial had detectable levels of DWV-A (90.0–92.7%) [32].

A subcollection of all *Varroa* collected were transferred to pupal hosts (*n* = 37) instead of being immediately frozen. In the *Varroa +* virus group, 6 out of 16 of these *Varroa* had detections of the tagged virus. One *Varroa* was lost and excluded. Three pupal hosts had detections of the tagged virus. Transmission from *Varroa* to pupal hosts was 17.6%, *n* = 17 [32].

### (b) Experiment 2: do *Varroa* acquire virus when feeding upon adult bees which previously cannibalized pupae?

**Figure 4.**
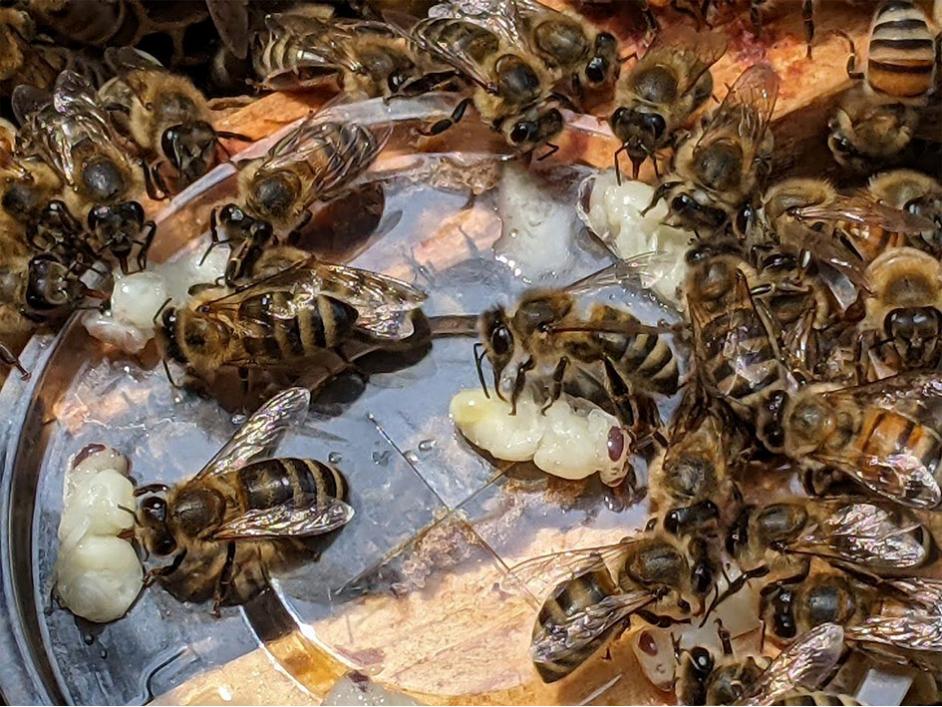
Worker bees cannibalize pupae. *Varroa,* 80.9%, in laboratory trials transmitted a tagged DWV after feeding on cannibal adult bees.

**Figure 5.**
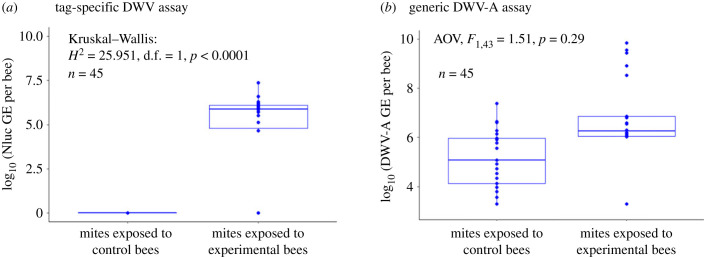
(*a*) Detections of NLuc nucleotide sequence in pupae which mites fed upon after removal from their adult bee host. *Varroa* were exposed to either control or experimental adult bees. *Varroa* which fed on adult bees that cannibalized infectious pupae were highly likely to later transmit the tagged virus acquired through adult feeding (80.9%, *n* = 20). (*b*) Detections of DWV-A in pupae which *Varroa* fed upon after removal from their adult bee host. There was no significant difference in natural DWV-A loads between control and experimental groups (AOV, *F*_1,43_ = 1.51, *p* = 0.29).

Naive *Varroa*, not previously exposed to tagged DWV-A, transmitted the tagged virus to a pupal host after feeding upon adult bees which had cannibalized infected pupae (figure 4). Of 21 staged *Varroa*, 17 (80.9%) successfully transmitted the acquired tagged virus to a naive bee host (figure 5). No detections of the novel nLuc insert were observed in the control groups (*n* = 23). There was no significant difference in wild-type DWV-A levels between experimentally exposed and control *groups* (AOV, *F*_1,43_ = 1.51, *p* = 0.29, table 2)

**Table 2.** Mean, median and s.d. of DWV-A and Nluc levels between experimentally exposed or control *Varroa*.

group	state	primer	mean	median	s.d.
overall DWV levels in pupae fed upon by exposed mites (all values in log10 GE per bee)					
experimental	naive	DWV	6.34	6.25	2.08
control	naive	DWV	5.85	5.77	2.23
negative control	naive	DWV	5.50	4.83	2.06
tagged DWV levels in pupae fed upon by exposed mites (all values in log10 GE per bee)					
experimental	naive	NLuc	4.60	5.86	2.60
control	naive	NLuc	0	0	0
negative control	naive	NLuc	0	0	0

### (c) Experiment 3: does DWV transmit indirectly from *Varroa*–*Varroa* on shared honeybee hosts?

**Figure 6.**
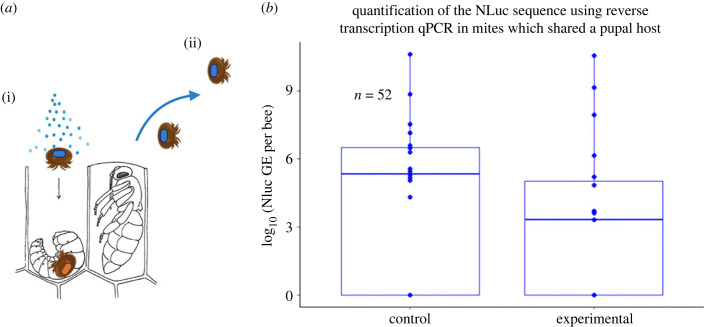
*Varroa*–*Varroa* virus transmission through shared honeybee host. (*a*) Experimental design. (i) Naive *Varroa* (orange mark) was inserted into a larval cells together with viruliferous *Varroa* (blue mark) carrying DWV-A NLuc. (ii) After 8 days the cells are uncapped, *Varroa* were removed and the levelss of tagged DWV in *Varroa* were quantified by the tag-specific qRT-PCR. (*b*) Quantification of the tagged DWV in *Varroa* by qRT-PCR by targeting NLuc sdequence. Results of the experimet suggest that naive *Varroa* may acquire DWV when sharing the same pupal host with a viruliferous *Varroa*. *Varroa* which were originally infectious did not have significantly higher levels of DWV-NLuc compared to the originally naïve *Varroa* which shared a host. Fewer naive *Varroa* transmitted the tagged virus than originally infectious *Varroa* which maintained the virus. (10/18 (55.56%) naive successfully transmitted versus 14/21 (66.67%) combined from both trials. (Pupal image credit: D G Mackean www.biology-resources.com, Created with BioRender.com.).

Naive *Varroa* acquired and subsequently transmitted a tagged virus while sharing a pupal host with another infectious *Varroa* (*n* = 20, 11/20 confirmed cases; figure 6). There was no significant difference in wild-type or clone-derived virus loads between *Varroa* which were originally naive or *Varroa* which were originally infectious after they shared a pupal host within the experimental group (AOV, *F*_1,37_ = 0.042, *p* = 0.84 and *F*_1,37_ *=* 1.70, *p* = 0.2). DWV-A loads in these *Varroa* averaged 8.09 log_10_ and 9.93 log_10_ GE per bee in the two trials, while their non-*Varroa* exposed counterparts averaged 4.47 log_10_ and 3.54 log_10_ GE suggesting *Varroa* infestations were causing covert infections (AOV, *F_1,136_ =* 17.55, *p* < 0.0001). Of the 11 *Varroa*, 4 acquired tagged virus (< 7 log_10_ GE/*Varroa*). *Varroa* which shared adult bee hosts were also shown to acquire the tagged virus (66.7% *n* = 6). All *Varroa* that originally were exposed to the tagged virus through pupal feedings had detectable levels of the NLuc reporter sequence, with a modest (but non-significant, AOV, *F_1,10_ =* 14.373, *p* = 0.10) reduction in virus load for the naive *Varroa* in mixed pairs. All *Varroa* in this trial had detectable levels of natural DWV-A (*n* = 18).

## 4. Discussion

*Varroa*, the honeybee and DWV offer a complex vector–host–pathogen system where the pathogen is both vector-borne and transmits communicably from host to host via multiple horizontal routes [8,9]. This provides many opportunities for hosts and vectors to shift from a susceptible to an infectious state as the virus circulates among members of the population. In a densely packed honeybee colony, infectious hosts can infect numerous nest-mates via social interactions, and the vectors which feed upon those hosts. In turn, infectious vectors can infect more hosts through their feeding. Here, we provide experimental evidence confirming several transmission routes in honeybee colonies and show their importance for the maintenance of DWV in both the vector and bee hosts. Most notably, we show transmission through horizontal routes between social hosts maintained the pathogen within the host population and then allowed for vectors in a susceptible state to later acquire and then transmit the pathogen.

We saw transmission of tagged virus between paired *Varroa* when feeding on the same bee host (Experiment 3). Vector to vector transmission of this sort is readily observed across arthropod vectors, most notably in ticks where co-feeding allows for non-systemic transmission between vectors [26,27]. Our work did not suggest that mite to mite transmission was possible with a non-viremic host, but rather confirmed that feeding on shared hosts by multiple mites allows for naive *Varroa* to acquire a virus because of the joint feeding with an infectious conspecific. Opportunities for indirect mite–mite transmission through a shared host may occur during both parts of the mite's life cycle. However, the greatest risk for acquiring DWV may occur during their non-reproductive phase when mites are freely moving from one bee to another to feed. During their non-reproductive phase mites move with a high frequency from host to host to feed [23]. Their active movement amongst the host population encourages opportunities for shared feedings or for naïve *Varroa* to feed on an infectious adult bee. We highlight this feature as particularly important for the acquisition of viruses by *Varroa*. Vectors which switch at the highest frequencies are most likely to acquire and subsequently transmit a pathogen [33].

In this dynamic system, adult bees can acquire infection through numerous non-vectored horizontal routes through their social interactions with other nest-mates. In Experiments 1 and 2, *Varroa* were both able to acquire and transmit DWV through two social behaviours: trophallaxis and cannibalization of pupae. Trophallactic interactions serve as a means of both communication and oral exchange of food between nest-mates [34], and are frequent in honeybee colonies [35]. Trophallaxis also aids in the maintenance of DWV by ensuring active transmission amongst adult nest-mates and has been established as an oral route amongst bees. However, the role that infectious hosts play in increasing the impacts of mite vectors has been poorly understood. Specifically, whether trophallaxis between infectious and susceptible nest-mates creates a bridge maintaining DWV for subsequent acquisition of the virus by a vector. Results from our Experiment 1 highlight the importance of these combined routes. Specifically, we were able to track viral transmission from introduction through multiple circulations between hosts and vectors. We show oral transmission of a tagged DWV occurred between donor and recipient bees when trophallaxis was encouraged between the two groups. *Varroa,* which fed upon recipient bees, acquired and subsequently vectored the tagged virus at low frequencies (figure 3). In this way, horizontal transmission of the virus through members of a social organization allowed for the maintenance and ultimate acquisition of the pathogen by a vector. We highlight this as a potentially important dynamic in this complex vector–host–pathogen triad.

We then asked if cannibalization, a social behaviour common in honeybees as a method of removing infested brood or in times of nutritional stress, could make adult worker bees an infectious reservoir of DWV to naïve mites which fed upon them. Results from Experiment 2 show *Varroa* which fed upon these bees subsequently vectored the novel DWV to new pupal hosts. These findings suggest that pupal cannibalization, which has been bred into honeybee populations as a form of behavioural hygiene [10], presents a possible risk factor for continued viral transmission within a honeybee colony. While cannibalization is a behavioural trait which provides benefits by reducing *Varroa* reproduction and survival [36], it is critical to assess, at the colony scale, the impacts of these opposing forces on colony health. Our results strengthen previous observations that cannibalization of infectious pupae transmits DWV to susceptible nest-mates [10], while also clearly showing an increase in infectious *Varroa* as an additional consequence of this social behaviour.

Combined our studies paint an important picture of viral circulation amongst infectious and susceptible members of a honeybee colony. More opportunities arise for *Varroa* to acquire DWV as infection spreads from host to host through multiple social behaviours.

DWV was present, but not prevalent, in honeybee populations before *Varroa destructor* jumped from *A. cerana* to *A. mellifera* [6,37,38]. Host-to-host transmission must have occurred to maintain DWV in honeybee populations prior to vectored transmission by *Varroa*. Vertical transmission of DWV from queen to progeny has long been attributed as the host to host pathway which successfully maintained DWV in honeybee populations [12]. Our results indicate that worker-to-worker transmission rates coupled with *Varroa* infestations may rival those from queen to offspring. Since workers are targeted by *Varroa* more often than queens [39], we would argue that worker-worker transmission, coupled with the concurrent vector transmission of *Varroa* as they move from bee to bee to feed, is the predominant means of maintaining DWV in colonies and puts these colonies at especially high risk when the vector arrives. Continued worldwide dispersal by *Varroa* supports this (i.e. when *Varroa* arrive in an area, DWV levels in worker honeybees tend to increase greatly and covary with *Varroa* infestation levels). When *Varroa* mites were introduced to the Hawaiian islands, viral loads in individual worker bees increased by a million-fold, establishing *Varroa*'s culpability in the emergence of DWV as a global pathogen [6]. The interplay of vector–host and host–host transmission for disease is rare in nature. In fact, most infectious diseases are either transmitted from host to host through non-vectored communicable routes, or vector borne, but very rarely both. Curiously, a select handful of emerging RNA viruses, including Zika, West Nile, Tembusu, salmon isavirus and Japanese encephalitis virus, are the exceptions to this rule [8,40–43]. Our results show that understanding this interplay can offer fundamental insights into routes of transmission and disease, and can point to management strategies that might break transmission, improving the health of a key pollinator.

## Data Availability

Supplementary data are available from Dryad Digital Repository [32].
